# Early Alterations of PACAP and VIP Expression in the Female Rat Brain Following Spinal Cord Injury

**DOI:** 10.1007/s12031-023-02151-w

**Published:** 2023-08-30

**Authors:** Sarah Thomas Broome, Mawj Mandwie, Catherine A. Gorrie, Giuseppe Musumeci, Rubina Marzagalli, Alessandro Castorina

**Affiliations:** 1 Laboratory of Cellular and Molecular Neuroscience (LCMN), School of Life Science, Faculty of Science, University of Technology Sydney, PO Box 123 Broadway, Sydney, NSW 2007 Australia; 2 Neural Injury Research Unit, School of Life Science, Faculty of Science, University of Technology Sydney, Sydney, NSW Australia; 3 Department of Biomedical and Biotechnological Sciences, Anatomy, Histology and Movement Sciences Section, School of Medicine, University of Catania, Catania, Italy

**Keywords:** Pituitary adenylate cyclase-activating polypeptide (PACAP), Vasoactive intestinal peptide (VIP), Spinal cord injury (SCI), Central nervous system (CNS)

## Abstract

Previous evidence shows that rapid changes occur in the brain following spinal cord injury (SCI). Here, we interrogated the expression of the neuropeptides pituitary adenylyl cyclase-activating peptide (PACAP), vasoactive intestinal peptides (VIP), and their binding receptors in the rat brain 24 h following SCI. Female Sprague-Dawley rats underwent thoracic laminectomy; half of the rats received a mild contusion injury at the level of the T10 vertebrate (SCI group); the other half underwent sham surgery (sham group). Twenty-four hours post-surgery, the hypothalamus, thalamus, amygdala, hippocampus (dorsal and ventral), prefrontal cortex, and periaqueductal gray were collected. PACAP, VIP, PAC1, VPAC1, and VPAC2 mRNA and protein levels were measured by real-time quantitative polymerase chain reaction and Western blot. In SCI rats, PACAP expression was increased in the hypothalamus (104–141% vs sham) and amygdala (138–350%), but downregulated in the thalamus (35–95%) and periaqueductal gray (58–68%). VIP expression was increased only in the thalamus (175–385%), with a reduction in the amygdala (51–68%), hippocampus (40–75%), and periaqueductal gray (74–76%). The expression of the PAC1 receptor was the least disturbed by SCI, with decrease expression in the ventral hippocampus (63–68%) only. The expression levels of VPAC1 and VPAC2 receptors were globally reduced, with more prominent reductions of VPAC1 vs VPAC2 in the amygdala (21–70%) and ventral hippocampus (72–75%). In addition, VPAC1 downregulation also extended to the dorsal hippocampus (69–70%). These findings demonstrate that as early as 24 h post-SCI, there are region-specific disruptions of PACAP, VIP, and related receptor transcript and protein levels in supraspinal regions controlling higher cognitive functions.

## Introduction

Pituitary adenylate cyclase-activating polypeptide (PACAP) and vasoactive intestinal peptide (VIP) are two closely related neuropeptides with shared receptors that mediate a diverse range of neuroprotective functions within both the central and peripheral nervous systems (Waschek 2013).

The activity of PACAP and VIP is mediated by three G-protein coupled receptors, PAC1, VPAC1, and VPAC2, which appear to regulate distinct biological functions (Hirabayashi et al. 2018). In general, VPAC1 and VPAC2 are implicated in most of the immune modulatory effects, while PAC1 receptors mainly promote neurotrophic and neuroprotective actions (Jansen et al. 2022). This aspect is particularly important for the interpretation of any therapeutic significance of the peptides. In fact, although all receptors bind with high affinity to both peptides, PAC1 displays a 100- to 1000-fold higher affinity for PACAP, compared to VIP (Ramos-Alvarez et al. 2015). Both peptides have been linked to a range of neurodegenerative and neuroinflammatory diseases, including spinal cord injury (SCI) (Jansen et al. 2022; Thomas Broome and Castorina 2022; Tsuchida et al. 2014; Van et al. 2021).

SCI is a devastating traumatic event that results in permanent neurological damage that is associated with significant social and economic disruption (Nakamoto et al. 2021). However, little is known about how SCI affects the brain, despite SCI resulting in long-term motor dysfunction, neuropathic pain, cognitive impairment, memory deficits, learning difficulties, and mood disorders (Li et al. 2020). These profound clinical impairments correlate with studies that show widespread and significant pathogenic alterations to the brain following SCI (Solstrand Dahlberg et al. 2018). Recently, it has been shown through magnetic resonance imaging (MRI) that alterations occur in the motor, sensory, and limbic systems following SCI, with pathological features demonstrating a reduction in myelin density, cortical atrophy, and consequent clinical impairments (Ziegler et al. 2018).

We have previously shown region-specific disruptions of GFAP and Iba1 in the brain within 24 h of SCI (Mandwie et al. 2022). These findings align with other evidence suggesting that SCI triggers signaling cascades that promote chronic neuroinflammatory states and inhibiting these pathways may improve cognition and mood in afflicted people (Li et al. 2020). However, the mechanisms behind this are unclear. In this context, only a few studies have investigated the role of PACAP and VIP in preclinical models of SCI, with the majority focusing on the local effects observed within the damaged spinal cord site. Dickinson and Fleetwood-Walker detailed how the expression of both peptides is markedly increased following peripheral axotomy or nerve ligation and can modulate neuropathic pain states following injury (Dickinson and Fleetwood-Walker 1999). Additionally, we have shown that both PACAP and VIP are increased following sciatic nerve constriction injury, a finding that correlated with the degree of behavioral disability in a subset of injured rats (Castorina et al. 2019). Likewise, in an impact injury model of traumatic brain injury—the impact acceleration model—it has been shown that PACAP exerted neuroprotective effects in the brain and in motor pathways, such as the corticospinal tract (Farkas et al. 2004; Tamás et al. 2006). Several studies have investigated traumatic-type injuries on both the spinal cord, the brain, and other central nervous system districts. For example, Atlasz et al. have shown that PACAP is a retinoprotective agent against a range of insults, including, excitotoxic, ischemic, and degenerative (Atlasz et al. 2010). Moreover, there have been a myriad of studies illustrating the anti-inflammatory and neuroprotective actions of these peptides that could contribute to their beneficial role in SCI (Carniglia et al. 2017; Gonzalez-Rey et al. 2010; Karunia et al. 2021; Masmoudi-Kouki et al. 2007). For example, PACAP and VIP have been shown to protect the hippocampus, prefrontal cortex, and amygdala from inflammatory insults (Mandwie et al. 2021; Marzagalli et al. 2016). These studies highlight the local immune modulatory and neuroprotective actions of the peptides. They also suggest that the endogenous PACAP/VIP system might play yet underappreciated roles as molecular substrates to help regain homeostatic control over the comorbid events triggered by SCI.

In this study, we sought to investigate the expression of PACAP and VIP and their related receptors within 24 h after SCI in the female rat brain. We analyzed gene and protein expression in distinct brain regions regulating cognitive and affective behaviors that are likely to be disrupted by devastating traumas such as SCI, including the hypothalamus, thalamus, amygdala, dorsal and ventral hippocampus, prefrontal cortex, and periaqueductal gray from rats that were exposed to experimental SCI and compared to sham controls 24 h after injury.

## Material and Methods

### Animals

All procedures were carried out with the approval of the University of Technology Sydney Animal Care and Ethics Committee (ETH13-0069), according to the guidelines set out by the National Health and Medical Research Council code of conduct for the use of animals in medical research. Twelve adult female rats (9 weeks old, body weight 250–300 g) were acquired from Animal Resource Centre (Perth, WA, Australia). Rats were housed in cages on a 12-h light-dark cycle with access to food and water ad libitum. Each cage was provided with environmental enrichment. Animals were randomly assigned to either mild contusion SCI group (SCI; *n* = 6) or sham surgery groups (sham; *n* = 6).

### Spinal Cord Injury

The surgical procedure has previously been described in detail (Mandwie et al. 2022). Briefly, rats were anesthetized with 4% isoflurane in 1 L/min oxygen and maintained at 2% isoflurane in 1 L/min oxygen. Local anesthetic (bupivacaine, 0.5%, 0.02 mL) was injected at surgical sites before an incision was made over the dorsal midline. A T10 laminectomy was performed to expose the spinal cord, and in the SCI group, animals were subject to a mild contusion injury (6.25 mm drop, 10 g weight) to the T10 spinal cord using the New York University impactor. The sham group were subject to a T10 laminectomy only without SCI. Following surgery, rats were allowed to recover in singly housed cages without environmental enrichment. Twenty-four hours later, rats were deeply anaesthetized and euthanized using pentobarbital sodium (100 mg/kg, i.p.). The brain of each rat was removed and snap frozen in liquid nitrogen. Brains were subsequently microdissected into the tissue blocks encompassing the following regions: prefrontal cortex, amygdala, dorsal and ventral hippocampus, hypothalamus, thalamus, and periaqueductal gray, as previously described (Mandwie et al. 2022).

### Real-Time Quantitative Polymerase Chain Reaction (RT-qPCR)

Total RNA was extracted with TRI reagent and precipitated with ice-cold 2-propanol following established protocols (Mandwie et al. 2021). Single-stranded cDNA was synthesized using the Tetro cDNA synthesis kit (Bioline, NSW, Australia) as per the manufacturer’s instructions. Real-time qPCR was performed to analyze the mRNA levels of the 5 genes listed in Table 1. The ribosomal protein 18S was used as the housekeeping gene (Table 1). Each PCR reaction consisted of 3 μL of 100 ng cDNA, 5 μL iTaq Universal SYBR Green Master Mix (Bio-Rad, NSW, Australia), and 0.8 μL of forward and reverse primers. Mean fold changes of each sample were calculated using the ΔΔCt method as previously described (Schmittgen and Livak 2008).

**Table 1:** List of primer sets used in real-time qPCR analyses. Forward and reverse primers were selected from the 5′ and 3′ region of each gene. The expected length of each amplicon is indicated in the right column

Accession #	Gene	Primer sequence (5′-3′)	Length (bp)
NM_009625.2	*Pacap*	Fwd 5′ CTGCGTGACGCTTACGCCCT 3′ Rev 5′ CCTAGGTTCTCCCCCGCGCC 3′	152
NM_011702.2	*Vip*	Fwd 5′ TGGCAAACGAATCAGCAGCAGCA 3′ Rev 5′ ACGCATTTGCTTTCTGAGGCGGG 3′	106
NM_007407.3	*Adcyap1r1*	Fwd 5′ CAGTCCCCAGACATGGGAGGCA 3′ Rev 5′ AGCGGGCCAGCCGTAGAGTA 3′	139
NM_011703.4	*Vipr1*	Fwd 5′ TCAATGGCGAGGTGCAGGCAG 3′ Rev 5′ TGTGTGCTGCACGAGACGCC 3′	127
NM_009511.2	*Vipr2*	Fwd 5′ GCGTCGGTGGTGCTGACCTG 3′ Rev 5′ ACACCGCTGCAGGCTCTCTGAT 3′	155
NM_011296.2	*S18*	Fwd 5′ CCCTGAGAAGTTCCAGCACA 3′ Rev 5′ GGTGAGGTCGATGTCTGCTT 3′	145

### Western Blot

Protein was extracted by homogenizing tissue in radioimmunoprecipitation assay (RIPA) buffer as previously described (Mandwie et al. 2021). The bicinchoninic acid assay (Pierce BCA Protein Assay Kit, ThermoFisher Scientific, VIC, Australia) was used to quantify protein. Thirty micrograms of protein was separated by SDS–polyacrylamide gel electrophoresis (SDS-PAGE) using 4–20% Mini-PROTEAN TGX Stain-Free Gels (15 well, Bio-Rad, NSW, Australia). The Precision Plus Protein Pre-stained Standard in All Blue molecular weight standard (Bio-Rad, NSW, Australia) was included for comparison and identification of bands of interest. Transfer to a PVDF membrane was performed using the semi-dry method (Trans-Blot Turbo Transfer System, Bio-Rad, NSW, Australia). Primary antibodies were incubated overnight in 5% skim milk in Tris-buffered saline with 0.1% Tween-20 (TBST) blocking solution at 4 °C. Membranes were incubated in secondary antibody for 1 h at RT. Antibodies and dilutions are summarized in Table 2. Membranes were visualized using chemiluminescence after 5-min incubation with Bio-Rad Clarity Western ECL Blotting Substrate Solution and images acquired using the Bio-Rad ChemiDoc MP System. Images were analyzed using Fiji ImageJ and ratios normalized to GAPDH, which was used as a loading control.

**Table 2:** Antibodies used in Western blots

Antibody	Dilution	Source (Cat. #)
Pituitary adenylate cyclase-activating polypeptide (PACAP)	1:1000	GeneTex (GTX37576)
Vasoactive intestinal peptide (VIP)	1:1000	GeneTex (GTX129461)
PAC1 receptor	1:1000	GeneTex (GTX30026)
VPAC1 receptor	1:1000	Sigma-Aldrich (SAB4503084)
VPAC2 receptor	1:1000	Abcam (ab2266)
Glyceraldehyde-3-phosphate dehydrogenase (GAPDH)	1:2000	Bio-Rad (VPA00187)
Goat Anti-Rabbit IgG HRP	1: 10,000	Bio-Rad (STAR208P)

### Statistical Analysis

All data is reported as mean ± SEM. Statistical analyses were calculated in GraphPad Prism Software v. 9.0.2. (GraphPad Software, La Jolla, CA, USA). Comparisons between two groups (sham vs SCI) were analyzed by unpaired *t* test. *p* values ≤ 0.05 were considered statistically significant.

## Results

### Pituitary Adenylate Cyclase Activating Polypeptide

**Fig. 1:**
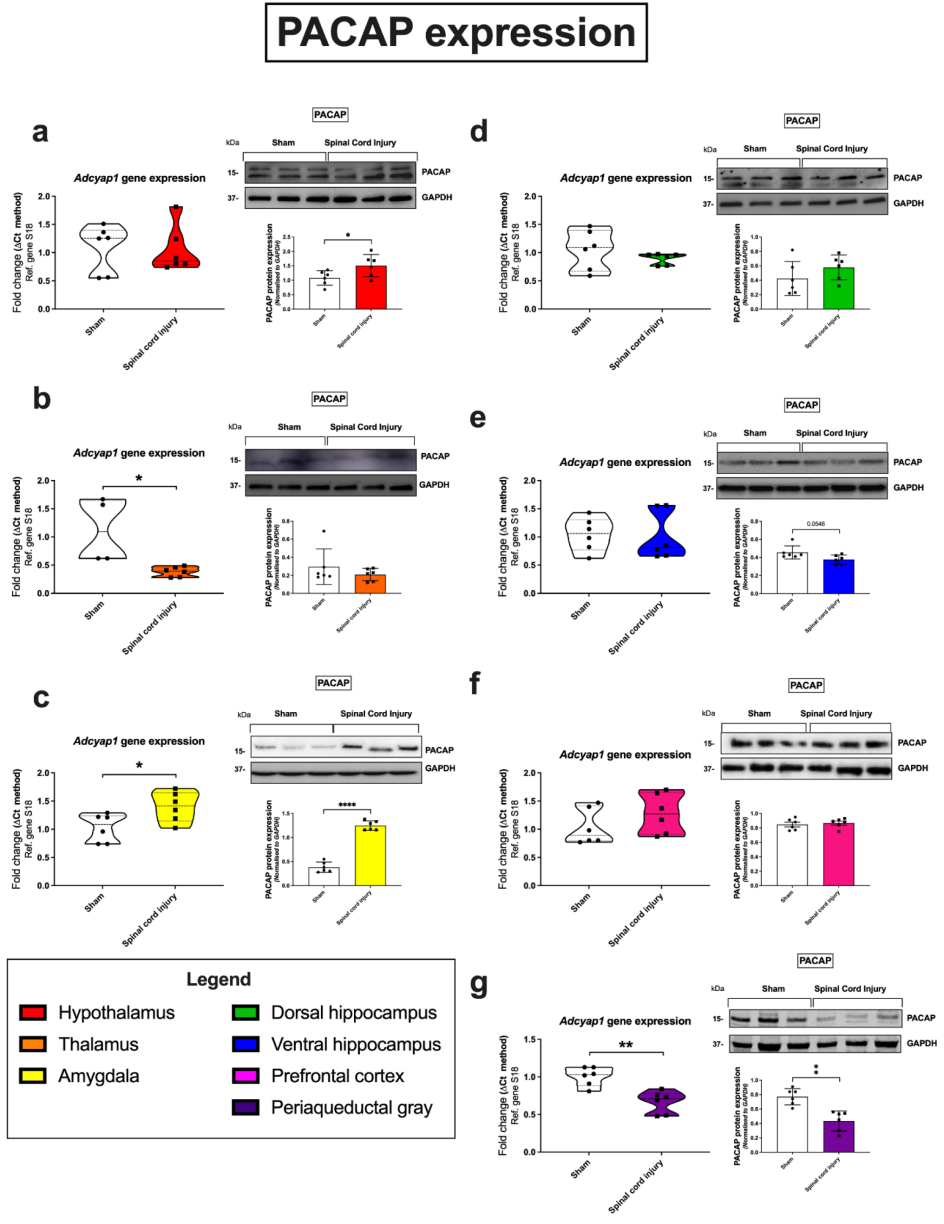
Gene and protein expression of the PACAP neuropeptide in distinct brain regions 24 h following SCI. Real-time qPCR analyses with representative Western blots and densitometry of PACAP expression in the hypothalamus (**a**), thalamus (**b**), amygdala (**c**), dorsal hippocampus (**d**), ventral hippocampus (**e**), prefrontal cortex (**f**), and periaqueductal gray (**g**) isolated from female rat brains subjected to SCI or sham surgery. Fold changes in gene expression were calculated using the ΔΔCt method after normalization to s18 (ribosomal protein s18), used as the housekeeping gene. Protein expression was normalized to GAPDH, the loading control. Densitometric results are expressed as the mean ± SEM from *n* = 4-6 rats per group. *p < 0.05 or **p < 0.01, compared to sham controls, as determined by unpaired Student’s *t* test. PACAP pituitary adenylate cyclase-activating peptide, s18 ribosomal protein s18, GAPDH glyceraldehyde-3-phosphate dehydrogenase

Twenty-four hours following SCI, the expression of both *Adcyap1* (*p = 0.0317; 138% of the sham group), the gene encoding PACAP peptide, and PACAP protein (****p < 0.0001; 350% of the sham group) was increased in the amygdala of animals that had received SCI, compared to sham controls (Fig. 1c). A similar trend was observed in the hypothalamus, in which PACAP peptide expression was significantly increased following SCI (*p = 0.045; 141% of the sham group; Fig. 1a), despite no changes in *Adcyap1* transcripts (104% of the sham group). Conversely in the thalamus, we observed a significant reduction in the expression of the *Adcyap1* gene (*p = 0.0135; 35% of the sham group; SCI vs sham; Fig. 1b), although this did not translate to changes in protein expression (95% of the sham group). PACAP expression was also downregulated in the periaqueductal gray at both transcript (**p = 0.0016; 68% of the sham group) and protein levels in SCI vs sham controls (**p = 0.001; 58% of the sham group; Fig. 1g). In the ventral hippocampus, despite SCI reducing PACAP protein expression, the change was not statistically significant (*p* = 0.0546 vs sham; 84% of the sham group; Fig. 1e).

### Vasoactive Intestinal Peptide

**Fig. 2:**
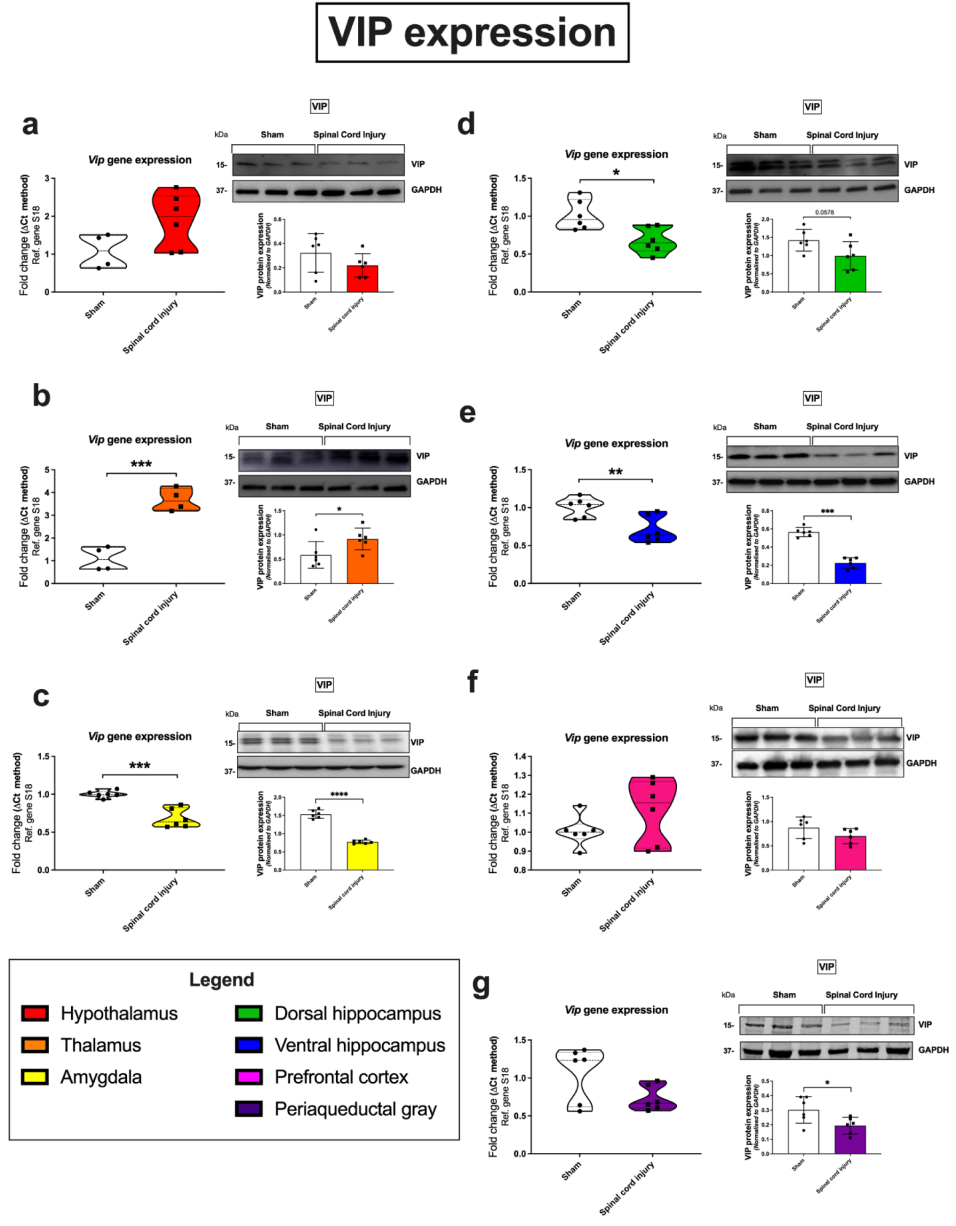
Gene and protein expression of the VIP neuropeptide in distinct brain regions 24 h following SCI. Real-time qPCR analyses with representative Western blots and densitometry of VIP expression in the hypothalamus (**a**), thalamus (**b**), amygdala (**c**), dorsal hippocampus (**d**), ventral hippocampus (**e**), prefrontal cortex (**f**), and periaqueductal gray (**g**) isolated from female rat brains subjected to SCI or sham surgery. Fold changes in gene expression were calculated using the ΔΔCt method after normalization to s18 (ribosomal protein s18). Protein expression was normalized to GAPDH, the loading control. Densitometric results are expressed as the mean ± SEM from *n* = 4-6 rats per group. *p < 0.05 or **p < 0.01, compared to sham controls, as determined by unpaired Student’s *t* test. VIP vasoactive intestinal peptide, s18 ribosomal protein s18, GAPDH glyceraldehyde-3-phosphate dehydrogenase

In the amygdala, both *Vip* gene expression (***p = 0.0001; 68% of the sham group) and protein (****p < 0.0001; 51% of the sham group) expression were significantly reduced in response to SCI, compared to sham controls (Fig. 2c). In the dorsal hippocampus, VIP was the only peptide that was altered following SCI (Fig. 2d). *Vip* transcripts were downregulated in response to SCI, compared to sham controls (*p = 0.0101; 67% of the sham group, Fig. 2d). This correlated with a decrease in protein expression of the peptide in the SCI group; however, this was not quite significant (*p* = 0.0578; 75% of the sham group, Fig. 2d). Similarly, VIP peptide was significantly reduced at both the mRNA (**p = 0.0073; 71% of the sham group SCI vs sham) and protein (****p < 0.0001; 40% of the sham group; SCI vs sham) levels in the ventral portion of the hippocampus (Fig. 2e). In the periaqueductal gray, VIP expression decreased significantly at the protein level (*p = 0.0340; 74% of the sham group), in the SCI group; however, the decrease was not significant at the transcriptional level (76% of the sham group; Fig. 2g). In contrast, an upregulation of both *Vip* gene expression (***p = 0.0004; 385% of the sham group) and VIP protein (*p = 0.0455; 175% of the sham group) expression was seen in the thalamus of SCI animals (vs sham; Fig. 2b).

### PAC1 Receptor

**Fig. 3:**
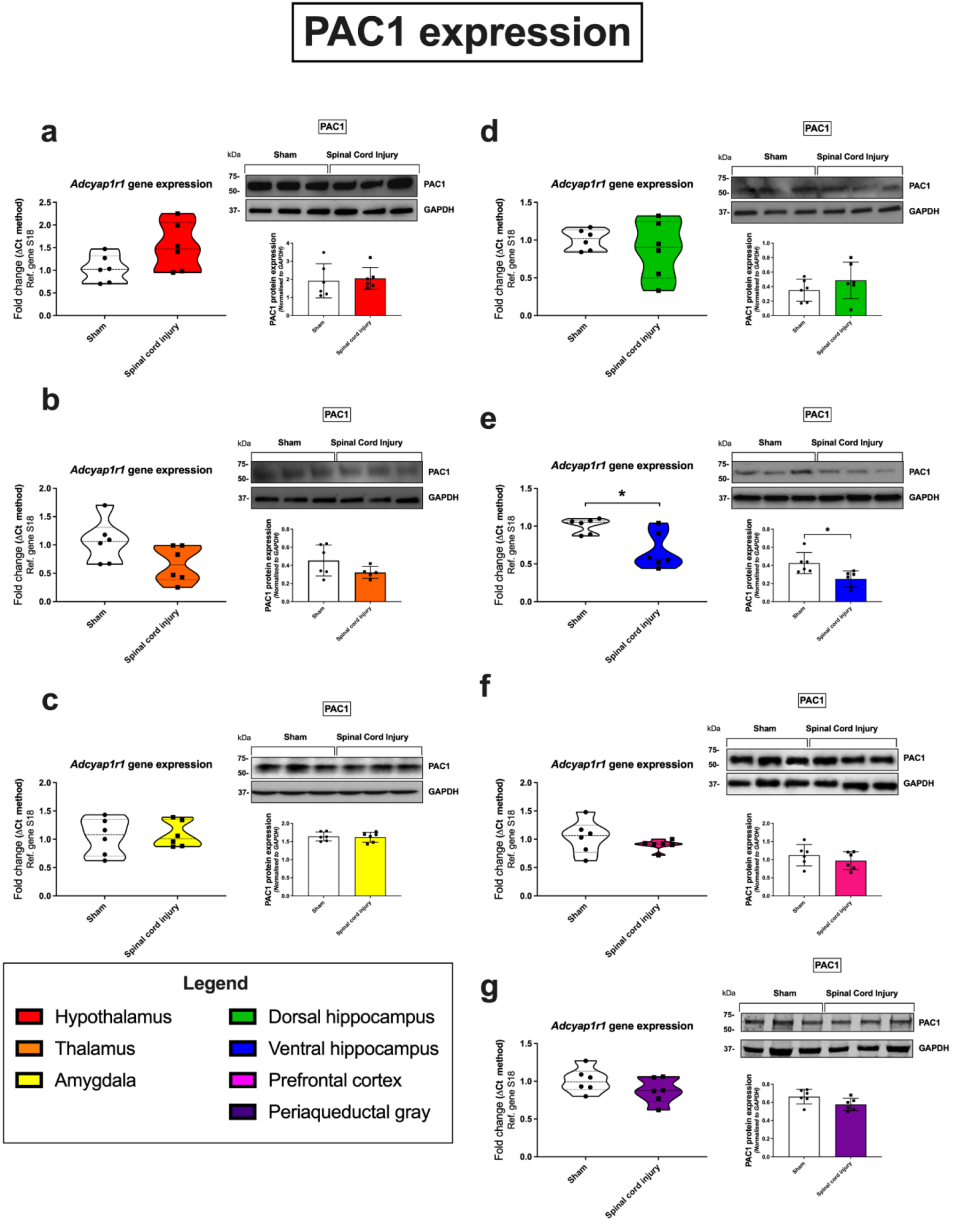
Gene and protein expression of the PAC1 receptor in distinct brain regions 24 h following SCI. Real-time qPCR analyses with representative Western blots and densitometry of PAC1 expression in the hypothalamus (**a**), thalamus (**b**), amygdala (**c**), dorsal hippocampus (**d**), ventral hippocampus (**e**), prefrontal cortex (**f**), and periaqueductal gray (**g**) isolated from female rat brains subjected to SCI or sham surgery. Fold changes in gene expression were calculated using the ΔΔCt method after normalization to s18 (ribosomal protein s18). Protein expression was normalized to GAPDH, the loading control. Densitometric results are expressed as the mean ± SEM from *n* = 6 rats per group. *p < 0.05 or **p < 0.01, compared to sham controls, as determined by unpaired Student’s *t* test. PAC1 PAC1 receptor, s18 ribosomal protein s18, GAPDH glyceraldehyde-3-phosphate dehydrogenase

The expression of PAC1 receptor was only significantly altered in the ventral hippocampus. Specifically, both the expression of *Adcyap1r1* mRNA (*p = 0.0104; 68% of the sham group) and expression of its protein product PAC1 (*p = 0.0149; 63% of the sham group) were decreased in SCI animals, compared to sham controls (Fig. 3e). There was also a marginal decrease of PAC1 expression within the thalamus, although this was not statistically significant (64% mRNA and 92% protein of the sham group; Fig. 3b).

### VPAC1 Receptor

**Fig. 4:**
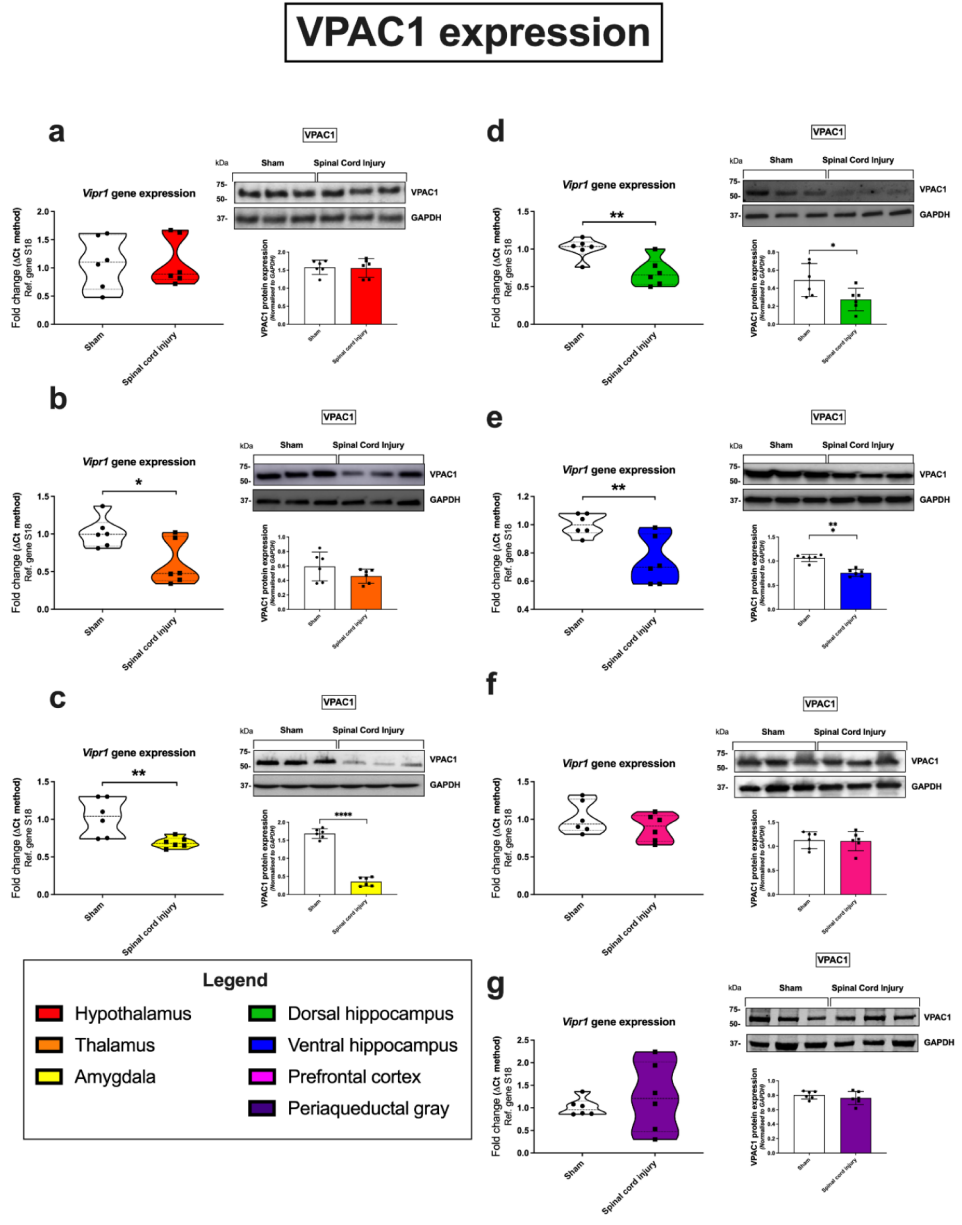
Gene and protein expression of the VPAC1 receptor in distinct brain regions 24 h following SCI. Real-time qPCR analyses with representative Western blots and densitometry of VPAC1 expression in the hypothalamus (**a**), thalamus (**b**), amygdala (**c**), dorsal hippocampus (**d**), ventral hippocampus (**e**), prefrontal cortex (**f**), and periaqueductal gray (**g**) isolated from female rat brains subjected to SCI or sham surgery. Fold changes in gene expression were calculated using the ΔΔCt method after normalization to s18 (ribosomal protein s18). Protein expression was normalized to GAPDH, the loading control. Densitometric results are expressed as the mean ± SEM from *n* = 6 rats per group. *p < 0.05 or **p < 0.01, compared to sham controls, as determined by unpaired Student’s *t* test. VPAC1 VPAC1 receptor, s18 ribosomal protein s18, GAPDH glyceraldehyde-3-phosphate dehydrogenase

Within the amygdala, SCI caused a downregulation of both *Vipr1* gene expression (**p = 0.0099; 70% of the sham group) and VPAC1 protein expression (****p < 0.0001; 21% of the sham group). In the dorsal hippocampus, both mRNA (**p = 0.0061; 70% of the sham group) and protein (*p = 0.0380; 69% of the sham group) levels were reduced in SCI animals (Fig. 4d). Similarly, the ventral hippocampus recorded the most significantly downregulation in SCI animals, both at mRNA (**p = 0.0069; 75% of the sham group) and protein levels (****p < 0.0001; 72% of the sham group) (Fig. 4e). Vipr1 transcripts were also significantly reduced in the thalamus (*p = 0.0191; 60% of the sham group; SCI vs sham; Fig. 4b), but this was not corroborated by protein expression data (*p* > 0.05; 85% of the sham group). *Vipr1* gene expression in the periaqueductal gray was not altered in response to SCI; however, we reported a broad distribution of results, reflecting individual variability in response across the sampled cohort of animals (124% mRNA and 95% protein of the sham group; Fig. 4g).

### VPAC2 Receptor

**Fig. 5:**
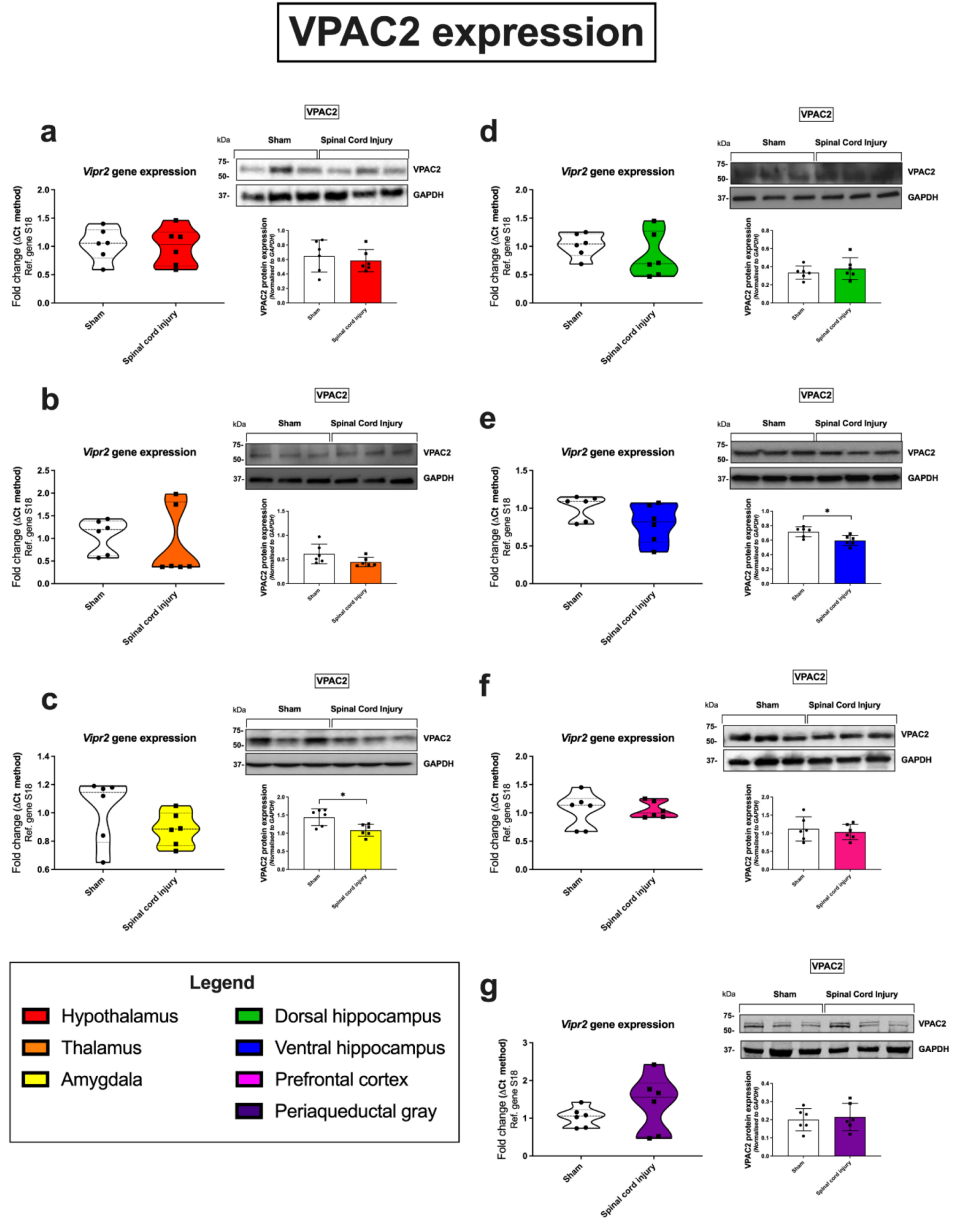
Gene and protein expression of the VPAC2 receptor in distinct brain regions 24 h following SCI. Real-time qPCR analyses with representative Western blots and densitometry of VPAC2 expression in the hypothalamus (**a**), thalamus (**b**), amygdala (**c**), dorsal hippocampus (**d**), ventral hippocampus (**e**), prefrontal cortex (**f**), and periaqueductal gray (**g**) isolated from female rat brains subjected to SCI or sham surgery. Fold changes in gene expression were calculated using the ΔΔCt method after normalization to s18 (ribosomal protein s18). Protein expression was normalized to GAPDH, the loading control. Densitometric results are expressed as the mean ± SEM from *n* = 6 rats per group. *p < 0.05 or **p < 0.01, compared to sham controls, as determined by unpaired Student’s *t* test. VPAC2 VPAC2 receptor, s18 ribosomal protein s18, GAPDH glyceraldehyde-3-phosphate dehydrogenase

SCI animals demonstrated a downregulation of VPAC2 protein expression in the amygdala (*p = 0.0115; 77% of the sham group; Fig. 5c). Similarly, VPAC2 also trended towards a reduction in the ventral hippocampus; however, this was only significant at the protein level (*p = 0.0137; 83% of the sham group; SCI vs sham; Fig. 5e). Similar to VPAC1, there is a large distribution of *Vipr2* gene expression in the periaqueductal gray of SCI animals (152% of the sham group; Fig. 5g).

## Discussion

There are only limited studies addressing CNS changes 24 h following SCI, and these are almost exclusively focused on the local spinal effects and using experimental models of ischemic spinal injury (Li et al. 2019a, b; Sönmez et al. 2007). A better understanding of both the molecular and functional changes that occur at supraspinal levels may be helpful for the development of prognostic and therapeutic tools for the management of traumatic SCI and associated comorbidities. In the present study, we revealed region-specific changes in the expression of the neuropeptides, PACAP and VIP, and their related receptors within 24 h of SCI in female rats. Experiments were specifically conducted in female rats as these are conventionally used in experimental SCI models due to their enhanced ability to recover and tolerate the surgical procedure compared with males (Datto et al. 2015). A further reason was related to ethical concerns, as in our experience, male rats subjected to SCI often develop a severe form of autotomy (self-mutilation) directed to their hind limbs and sometimes genitalia that we have never observed in female rats. The exaggerated autotomy seen in male SCI rats also seems to occur after neurectomy of peripheral nerves, as shown in other studies (Wagner et al. 1995). To the best of our knowledge, this is the first study to provide evidence of molecular alterations of this neuropeptide system in the brain following traumatic SCI.

The neuroprotective and immunomodulatory actions of PACAP and VIP are well established (Abad and Tan 2018; Waschek 2013). Studies in PACAP-knockout (KO) mice have shown that a loss of PACAP results in age-related degenerative signs earlier than wild-type animals, with the appearance of overt signs of increased neuronal vulnerability, systemic degeneration, and increased inflammation, highlighting the importance of this neuropeptide in the maintenance of a healthy CNS (Reglodi et al. 2018). In contrast, VIP has been linked to most of the neuropeptide immunomodulatory actions and has been explored as a potential anti-inflammatory target in animal models of Parkinson’s disease, where a single intracerebroventricular injection of VIP was able to reduce microglial activation and prevented neurodegeneration (Delgado and Ganea 2003).

In the context of SCI, most studies have been focused on investigating molecular changes occurring at the site of injury—that is, within the spinal cord. Furthermore, the large bulk of investigations attempting to describe the contribution of this neuropeptides’ family have been restricted to PACAP and not VIP nor the receptors. Nonetheless, these studies have offered the opportunity to comprehend some of the beneficial properties of these neuropeptides in the injured CNS. For example, in a contusion model of SCI, heterozygous PACAP-KO mice displayed impaired motor function and greater neuronal cell death compared to wild-type mice (Tsuchikawa et al. 2012). Moreover, when PACAP was delivered immediately after SCI, motor recovery was significantly improved and this was associated with increased axonal regeneration compared to saline-treated controls, suggesting that PACAP stimulates functional recovery by promoting neuroregeneration and/or neuroplasticity within the spinal cord (Tsuchida et al. 2014). These results align with another SCI study, in which it was shown that blocking BDNF signaling after SCI inhibits neurological function recovery (Li et al. 2019a, b). There has long been a connection with PACAP and the neurotrophic factor BDNF. For instance, in prior work, we have shown that in Schwann cells—the peripheral myelin-producing glia—BDNF treatment mimicked PACAP actions (Castorina et al. 2015). Another study has shown that injury-associated endogenous BDNF induces PACAP expression in rat sensory and motor neurons (Pettersson et al. 2014). The close relationship between BDNF and PACAP is important as BDNF has been extensively researched in the context of SCI and has been found to be involved in functional recovery and neuropathic pain (Garraway and Huie 2016). Despite the lack of VIP studies in SCI, there are some studies in models of peripheral nerve injuries that demonstrate how VIP is also able to promote remyelination and attenuate the inflammatory burden within the distal nerve stump following a nerve injury (Woodley et al. 2019). Additionally, we have previously demonstrated that, following sciatic nerve constriction injury, VIP expression is abnormally increased in the periaqueductal gray, and this correlated with the development of a persistent state of behavioral disability (Castorina et al. 2019). The focus on PACAP over VIP in the context of SCI is surprising, especially since we reveal that VIP and VPAC1 were most significantly altered in the brain 24 h after SCI. This could be due to an early inflammatory response in the brain prior to any obvious neuronal changes (Jure and Labombarda 2017).

VIP and VPAC receptors are linked to the peptide’s immunomodulatory functions, suggesting that the disruptions observed in their expression could be linked to inflammation. It is known that injuries to the spinal cord trigger pathological signaling cascades that result in microglial and astrocyte activation and, consequently, neuroinflammation (Li et al. 2020). In addition, we have previously shown that the expression of both astrocytes and microglia activation markers (GFAP and Iba1, respectively) is altered 24 h after SCI (Mandwie et al. 2022). Interestingly, there were no similarities in the pattern of gene and protein expression of neuropeptides and the aforementioned inflammatory markers. The only noteworthy observation is related to the amygdala, where the robust GFAP upregulation was associated with significantly reduced expression of VIP and both VPAC receptor subtypes in this study. However, Faden et al. have proposed that it is chronic neuroinflammation in the brain that occurs following SCI, which subsequently may lead to the neurodegeneration of CNS regions not directly affected by the trauma but responsible for the comorbid cognitive and affective decline (Faden et al. 2016). These evidences would explain the disparity between the inflammatory changes seen in several CNS regions versus the subtler changes in neuropeptides’ expression profiles, since SCI occurred only 24 h prior to tissue harvest, a time window that may not sufficient to record molecular changes to suggest central neurons deterioration. While there is no clear evidence of a solid link between the rapid adaptive changes in the brain regulation of this neuropeptide system and the previously observed region-specific inflammatory profile in the SCI brain, further studies are required to elucidate the exact mechanisms behind these disruptions.

Nonetheless, we demonstrated significant changes in neuropeptide expression in the amygdala and ventral hippocampus. This aligns with the fact that most SCI cases are traumatic experiences, caused by traffic accidents, violence, sports, and/or falls (Alizadeh et al. 2019). The amygdala processes fearful and threatening stimuli and participates in the formation of new memories associated with the fearful/traumatic experience (Baxter and Croxson 2012). The ventral hippocampus is mainly involved in stress and emotion and is linked to stress circuits via its tight relationships with the amygdala (Fanselow and Dong 2010). The dramatic changes observed in these regions suggest that the traumatic experience during SCI might be responsible for the immediate changes in the amygdala and hippocampus in view of their involvement in the processing of these emotional and cognitive responses. This could explain why other regions, known to be affected by SCI, demonstrated little or no change in neuropeptide expression profile, at least immediately after injury. For instance, neuropathic pain is often a common side effect of SCI. We analyzed the neuropeptide profile across several nociceptive regions, including the periaqueductal gray, the prefrontal cortex, and hypothalamus (Garland 2012). Perhaps, the expression profile of PACAP and VIP is altered at time points further from the initial injury, since it has been shown that PACAP has a role in nociception (Jongsma et al. 2000), with PAC1 knockout mice exhibiting a 75% reduction in nociceptive responses, providing evidence of a role of the PACAP/PAC1 axis in the mediation of nociceptive responses (Jongsma et al. 2001). Future studies at later time intervals could be useful to determine the involvement of this neuropeptide system in regulating other higher cognitive functions associated with the comorbidities of SCI.

The pleiotropic nature of these peptides justifies the broad involvement of PACAP and VIP in several neuropathological processes, many of which may require several days to establish. Additionally, these studies should be performed with both male and female rats due to the known sexual dimorphism that exists in neurological pathologies. It has been shown that female rats benefit from their innate heightened immune activities, which facilitate spontaneous recovery from CNS injury (Hauben et al. 2002). In SCI, functional recovery also shows some degree of sexual dichotomy in mice, with young adult female mice displaying enhanced neurological recovery compared to males (Li et al. 2022).

Nardone et al. reported that SCI leads to a wide range of sensorimotor and autonomic nerve damage and this results in the rapid reorganization of supraspinal structures within hours of peripheral nerve damage (Nardone et al. 2018). Knowing where this reorganization occurs can help predict long-term outcomes for SCI patients. A survey of people with SCI revealed that depression, anxiety, psychological trauma, and pain were among the most severe symptoms associated with SCI (Tulsky et al. 2015). This aligns with our data, which showed pronounced disruptions in the amygdala and ventral hippocampus, areas commonly affected by traumatic events. Moreover, this reorganization can influence how we treat and manage SCI. For example, PACAP has been suggested as a neuroprotective therapeutic and peripheral administration of the peptide requires the peptide transport system (PTS)-6 to cross the blood–brain barrier (Tamas et al. 2012). It has been shown that SCI results in regional and temporal-specific changes in PTS-6 that could affect the delivery of PACAP to the CNS (Banks et al. 1998). Additionally, recognizing the brain regions specifically affected by SCI can help in the long-term management of neurological comorbidities associated with this devastating condition.

## Conclusion

We provide evidence that the expression of the neuropeptides, PACAP and VIP, along with their related receptors is altered 24 h after SCI in a region-specific manner (results are summarized in Table 3). Interestingly, it is VIP and VPAC1 that are most strongly affected. VIP and VPAC receptors are more closely associated with modulatory activities on the CNS inflammasome than PACAP and PAC1, suggesting a link with the neuroinflammatory cascades triggered by SCI. Of note, the amygdala and ventral hippocampus exhibited the most disruptions of these molecular targets, pinpointing how SCI can trigger early perturbations in fear/stress pathways, promote the generation of trauma-related memories, and/or facilitate the establishment of anxiety and/or other affective disturbances. Therefore, although further studies are needed to determine both the early and long-term consequences of these changes on neuronal activity to define the potential implications in the development of comorbid behavioral alterations, this study emphasizes the need for SCI research to also focus on supraspinal structures with the aim to develop strategies aimed to rescuing several of the comorbidities arising from SCI and, perhaps, the associated peripheral neuropathies.

**Table 3:** Summary of the disruptions in the mRNA and protein expression of PACAP, VIP, and related receptors at 24 h after SCI

Brain region	PACAP		VIP		PAC1		VPAC1		VPAC2	
	Gene	Protein	Gene	Protein	Gene	Protein	Gene	Protein	Gene	Protein
Hypothalamus	104	141	161	124	161	132	115	100	98	104
Thalamus	35	95	385	175	64	92	60	85	119	80
Amygdala	138	350	68	51	109	99	70	21	91	77
Dorsal hippocampus	96	164	67	75	85	142	70	69	84	119
Ventral hippocampus	104	84	71	40	68	63	75	72	80	83
Prefrontal cortex	127	103	112	86	93	96	88	100	107	98
Periaqueductal gray	68	58	76	74	87	88	124	95	152	112

Changes (calculated as percent of sham) are reported

*N.C.* no change, *PACAP* pituitary adenylate cyclase-activating polypeptide, *VIP* vasoactive intestinal peptide, *PAC1* PAC1 receptor, *VPAC1/2* VPAC1/2 receptor

## Data Availability

All data generated or analyzed in this study has been included in this published article. Raw data can be made available upon reasonable request to authors.
